# Optimizing an automated sleep detection algorithm using wrist-worn accelerometer data for individuals with chronic pain

**DOI:** 10.1371/journal.pone.0319348

**Published:** 2025-04-04

**Authors:** Louis Faust, Emma Fortune, Omid Jahanian, Sey Oloyede, Clifford Trouard, Suzanne Dixon, Erica Torres, Chris Sletten, Paul Scholten

**Affiliations:** 1 Robert D. and Patricia E. Kern Center for the Science of Health Care Delivery, Mayo Clinic, Rochester, Minnesota, United States of America; 2 Rehabilitation Medicine Center, Mayo Clinic, Rochester, Minnesota, United States of America; 3 Pain Rehabilitation Center, Mayo Clinic, Jacksonville, Florda, United States of America; 4 Department of Physical Medicine & Rehabilitation, Mayo Clinic, Rochester, Minnesota; Portugal Football School, Portuguese Football Federation, PORTUGAL

## Abstract

**Objective:**

To optimize a wrist-worn accelerometer-based, automated sleep detection methodology for chronic pain populations.

**Patients and methods:**

A cohort of 16 patients with chronic pain underwent free-living observation for one week before participating in an Interdisciplinary Pain Management Program. Patients wore ActiGraph GT9X devices and maintained a sleep diary, documenting their nightly bedtimes and wake times. To derive sleep quality measures from accelerometry data, the Tudor-Locke sleep detection algorithm was employed. However, this algorithm had not been validated for chronic pain patients. Therefore, a sensitivity analysis of the algorithm’s parameters was conducted, identifying a set of parameters which maximized the agreement between sleep periods identified by the algorithm and sleep periods identified by participant’s sleep logs, which were considered ground truth. Sleep measures derived when using the optimized parameters were then compared against sleep measures derived using the default parameters.

**Results:**

Our optimized parameter set achieved a mean sleep detection agreement of 67% with participant’s sleep logs, while the default parameter set achieved a mean agreement of 50%. Statistically significant differences were observed between sleep measures from the optimal and default parameter sets (P *< *.001). These findings suggest an optimized parameter set should be favored for chronic pain populations.

**Conclusion:**

The Tudor-Locke algorithm provides automated sleep detection for accelerometry data; however, caution must be exercised when applying the algorithm to populations beyond its validated scope. In this manuscript, we provide an empirically optimized parameter set for applying this algorithm to adults with chronic pain.

## Introduction

The bidirectional relationship between sleep and chronic pain necessitates monitoring sleep in chronic pain populations [1]. Poor sleep quality disrupts pain modulation, increasing pain sensitivity and reducing tolerance [2–5]. Conversely, chronic pain disrupts sleep, resulting in difficulty in falling asleep, staying asleep, and achieving restorative sleep [6]. These disruptions may stem from pain-related discomfort or nocturnal awakenings due to pain flare-ups [7]. Poor sleep quality is also linked to functional impairments, reduced quality of life, and higher risks of depression and anxiety [8–10]. Therefore, improving sleep outcomes is an essential pillar of pain management [11].

To comprehensively assess sleep in chronic pain populations, various methods have been employed, including subjective questionnaires and polysomnography (PSG): recognized as the gold standard for evaluating sleep architecture and diagnosing sleep disorders [7,12]. While PSG provides accurate measurements, its reliance on overnight hospital stays limits scalability, and its controlled setting may not fully reflect natural sleep patterns [13,14]. Therefore, at-home monitoring tools may provide more ecologically valid assessments of sleep quality [15].

Accelerometers offer these capabilities as non-invasive tools that provide high-resolution, objective sleep data which correlate well with PSG, making them preferable to self-report surveys [16–19]. As such, accelerometers have been used to study sleep across various populations, including those with sleep disorders and chronic pain [20–22]. However, significant preprocessing is required to accurately extract relevant measures, demanding careful methodological choices [23,24].

To simplify preprocessing, several software platforms offer user-friendly interfaces for computing measures of physical activity, sleep, and device wear time. Despite their convenience, users must still understand and select the appropriate methods to ensure reliable outcomes. For example, ActiGraph’s ActiLife software uses a three-stage process: (1) an algorithm classifies each raw data epoch as “sleep” or “wake”, (2) using the resulting sleep/wake time series, a different algorithm then identifies sleep periods, lastly (3) sleep measures, such as duration and efficiency, are computed for each identified sleep period. The user must choose algorithms for stages (1) and (2) of this process, which can significantly impact results [25,26].

This study focused on stage (2), specifically investigating the Tudor-Locke (TL) algorithm, a method for identifying sleep periods from sleep/wake time series [27]. While the TL algorithm has been validated for children, its suitability for populations beyond this scope, such as those with chronic pain likely to exhibit abnormal sleep characteristics, remains unclear [27,28]. Therefore, this manuscript aimed to optimize the TL algorithm for a cohort of chronic pain patients. A sensitivity analysis identified a set of parameter values that maximized the agreement between the algorithm and participants’ self-reported sleep logs, providing evidence for a new set of parameter values when examining chronic pain populations.

## Materials and methods

### Study design

This study focused on a cohort of chronic pain patients who participated in an interdisciplinary pain management program (IPMP) at Mayo Clinic. Patients in this program have “high-impact” chronic pain that significantly impacts their physical and often emotional functioning [29]. Typically, these patients have persistent pain irrespective of the underlying etiology, despite extensive treatment with usual interventions including physical therapy, medications, interventional pain treatments and, in some cases, surgery. The overall aim of our research is to evaluate the efficacy of IPMP on patient’s pain management, utilizing objective measurements of physical activity and sleep to assess participants’ treatment response. The aim of the study presented in this manuscript focused on optimizing the TL sleep period identification algorithm to characterize sleep in chronic pain patients, pre-IPMP participation.

Participants were eligible for this study if they met the following qualifications: 1) were between 18-85 years old; 2) were enrolled in the IPMP for chronic pain; 3) agreed to wear an activity monitor; and 4) agreed to complete follow-up assessments. Participants were *ineligible* if any of the following criteria applied: 1) were receiving worker’s compensation; 2) could not provide independent consent; 3) were pregnant or became pregnant during the study time frame; 4) were employed at Mayo Clinic; 5) were non-independent ambulators; or 6) had a functional movement disorder.

Upon enrollment, participants were mailed an ActiGraph GT9X device to wear for one week before starting the IPMP and instructed to perform their typical daily activities and nightly sleep routines. Participant’s baseline sleep routines were captured using ActiGraph devices and self-report sleep diaries. ActiGraphs were set to a sampling rate of 30Hz. Sleep measures were processed using ActiLife software (v6.13.4) and R (v4.3.1). Statistical analyses were conducted using R (v4.3.1) and Python (v3.12.1).

To ensure sleep routines were reliably represented, participants needed at least five nights of sleep in which 1) the device was worn to bed and 2) sleep and wake times were documented in a sleep log. This five night minimum has been shown to reliably estimate weekly average sleep duration and variability [30]. Additionally, recorded nights did not need to be consecutively measured [30]. Among the 17 participants assessed at baseline, 16 had sufficient wear time and sleep log data. The median number of eligible nights was 7 (IQR, [6, 7]).

### Ethics

This observational study was approved by the Mayo Clinic’s IRB after a full board review under protocol number 21-006718. All participants provided written informed consent prior to taking part in the study. The study ran from September 19th, 2022, through December 5th, 2023.

### Data processing

To derive sleep measures from raw accelerometry data, ActiLife employs a three-stage process. The first stage requires an algorithm to process the raw accelerometry data, classifying every epoch as “sleep” or “wake”. In this study, “epochs” were one minute long, as required by the TL algorithm for detecting sleep periods later in stage two [31]. ActiLife provides two algorithms for stage one: Cole-Kripke and Sadeh. Cole-Kripke was chosen over Sadeh as it was developed and validated on an adult population [32], while Sadeh was validated on children and young adults [33]. Although ActiLife also allows users to implement their own custom algorithm for this stage, Cole-Kripke’s comparable performance to state-of-the-art algorithms and built-in availability, makes it the pragmatic choice for clinical use [34].

In the second stage, a different algorithm identifies sleep periods from the resulting sleep/wake time series. ActiLife offers two options: the TL algorithm and ActiLife’s own algorithm. Sleep periods can also be manually defined using self-reported sleep logs.

The TL algorithm uses three parameters to detect sleep periods: (*1*) *Bedtime Definition* - the minimum number of consecutive sleep epochs necessary to start a sleep period (default: 5 epochs); (*2*) *Wake Time Definition* - the minimum number of consecutive wake epochs which mark the end of a sleep period (default: 10 epochs); and (*3*) *Min Sleep Period Length* - the minimum amount of time necessary between the bedtime and wake time (default: 160 minutes). The TL algorithm then parses the sleep/wake epochs to identify sleep periods as contiguous blocks of epochs fitting within these definitions. In addition, ActiLife provides two optional parameters. *Minimum Non-Zero Epochs*, which requires the sleep period to contain at least *X* epochs with activity counts greater than zero to prevent falsely detecting non-wear periods as sleep (default: 0 epochs). And *Max Sleep Period Length*, setting an upper limit to sleep period length. By default, this is set to 1440 minutes, as ActiLife restricts sleep periods to less than 24 hours [31].

For completeness, ActiGraph’s own sleep period detection algorithm was evaluated. However, since it does not require tuning parameters, a sensitivity analysis was not necessary.

The final stage computes sleep measures from each identified sleep period, requiring no user input.

### Tudor-Locke sensitivity analysis

To identify optimal TL parameters for our chronic pain population, we conducted a sensitivity analysis on four parameters: *Bedtime Definition*, *Wake Time Definition*, *Min Sleep Period Length*, and *Minimum Non-Zero Epochs*. *Max Sleep Period Length* was kept at its default value to avoid arbitrary upper limits on sleep period length. The values evaluated were:

- *Bedtime Definition* and *Wake Time Definition*: 5, 10, 15, 20, 25, and 30 minutes
- *Min Sleep Period Length*: 30, 60, 90, 120, 150, 160, and 180 minutes
- *Minimum Non-Zero Epochs*: 0, 5, 10, 15, 20, and 25 epochs

Using all possible combinations of these parameters, we tested 1,512 unique parameter sets, opting for a code-based approach using the R package **‘**actigraph.sleepr**’** to avoid manually operating ActiLife’s graphical user interface. This package includes implementations of the Cole-Kripke and Tudor-Locke algorithms [35].

Each parameter set’s performance was measured by the Percentage of Agreement between TL algorithm-derived sleep periods and participant’s sleep logs. Percentage of Agreement was calculated as the number of one-minute time-points where both methods agreed the participant was asleep, divided by the total number of unique time-points where *either* method classified the participant as asleep.

As our focus centered on agreement between sleep periods, periods of wake were not included. Additionally, periods of wake/restlessness *within* a sleep period were not evaluated, as they were not recorded in sleep logs. Although we considered metrics like Cohen’s Kappa for inter-rater reliability, we found Percentage of Agreement more suitable, as it imposed a greater penalty when the TL algorithm failed to identify any sleep periods for a night. Such failures are often due to excessive movements throughout the sleep period. These movements result in an insufficient number of sleep epochs—identified by the Cole-Kripke algorithm—from occurring consecutively, preventing a sleep period from being recognized by the TL algorithm for that night.

For each parameter set, Percentage of Agreement was calculated separately for each participant and then averaged across all participants. This approach limited bias from the varying amounts of data contributed by participants. The optimal parameter set was selected based on the maximum average agreement score.

### Sleep measure comparison between default and optimized Tudor-Locke parameters

After identifying an optimal parameter set for our chronic pain population, we evaluated the TL algorithm with this optimized parameter set (TLO) through two analyses, comparing TLO against the TL algorithm with default parameters (TLD) and participant’s sleep logs (SL).

Sleep measures derived from each method were compared, with SL considered as ground truth. All ActiLife sleep measures were included (detailed in Table 1). Note that *Latency* - the time it takes to fall asleep, is included for completeness. However, the TL algorithm will always produce a value of zero, as the algorithm requires the first epoch of any sleep period to be a sleep state [31].

**Table 1:** The set of sleep measures computed by ActiLife, including definitions for each measure and the aggregation methods utilized when multiple sleep periods were detected within the same day.

Sleep measure	Definition	Daily aggregation method
Latency	Number of wake epochs between bedtime and the first sleep epoch	Summed from all sleep periods
Efficiency	Total sleep time divided by total duration of sleep period	Sum of total sleep time from all periods, divided by the sum of total duration of all sleep periods
Total Minutes in Bed	Total time in bed	Summed from all sleep periods
Total Sleep Time	Total time spent asleep	Summed from all sleep periods
Wake After Sleep Onset (WASO)	Total time spent awake after first sleep epoch	Summed from all sleep periods
Number of Awakenings	Number of unique, contiguous blocks of wake epochs	Summed from all sleep periods
Average Awakening Length	Average length of a block of wake epochs, “block” could refer to a single epoch	Average length of all awakenings across sleep periods
Movement Index	Total number of epochs with one or more activity counts, divided by total time in bed [36,37].	Count of active epochs, divided by total minutes in bed (epochs taken from all sleep periods, total minuted in bed summed from all sleep periods) [active epoch = epoch with activity count *≥ * 1]
Fragmentation Index	Number of times sleep was terminated after one minute, divided by total sleep time [37,38].	Count of one minute sleep epochs divided by count of sleep segments (epochs taken from all sleep periods)
Sleep Fragmentation Index	Quantifies restlessness during sleep by summing the Fragmentation index and Movement index [37,39].	Movement index summed from all sleep periods + Fragmentation index summed from all sleep periods

Sleep measures were calculated for each detected sleep period, typically resulting in one set per night. However, if multiple periods were detected within the same night, the measures were aggregated by day to allow for one-to-one comparisons across methods (see Table 1 for details). All sleep periods were assigned to the day they *ended*.

Sleep measures were calculated from detected sleep periods, leading to missing data on days without detected periods. To address this, a complete-case analysis was applied where possible [40]. A supplementary analysis compared TLD and TLO using both complete-case and zero-imputation methods to assess any potential bias (see Section S1 in the Supplementary Materials). No bias was observed resulting from the choice of missing data strategy [40,41].

Two comparative analyses were conducted:

- **Extended Bland-Altman plots**: An extension to the original Bland-Altman plots allowing for repeated measures within subjects [42] was used to compare SL (considered the gold standard for this analysis) with sleep periods detected from TL, employing a complete-case strategy when necessary.
- **Wilcoxon Signed-Rank Test**: We tested for significant differences in sleep measures between SL and TLD, SL and TLO, and TLD and TLO, respectively, using participants’ mean sleep measures as paired values. Statistical significance was defined at a *P* value of 0.05 [43].

Finally, visualizations and descriptive statistics are provided, including side-by-side box plots and tables showing means and standard deviations. Missing sleep measures were excluded, with distributions representing all detected sleep periods. A supplementary analysis was performed in which sleep measures were compared among participants stratified by age: ≤ 50 years and > 50 years, with complete details available in Section S6 of the Supplementary Materials.

## Results

### Participant population

The study included 16 adults with chronic pain. The median participant age was 49.5 (IQR, [33, 56]) and 87.5% were women. All participants identified as “White/Caucasian” and “Not Hispanic or Latino”. The median participant BMI was 29 (IQR, [24, 30.8]), 50% reported an annual household income ≥ $90,000. Among these patients, 81.25% had at least some college education, with 18.75% having obtained a Master’s degree. Participants had a mean duration of pain of 9.99 years (SD =  8.73), mean severity of pain, as measured by pain numeric rating scale (NRS), of 6.38 (SD =  2.03) [45], SF-36 (quality of life measure) for pain of 30.62 (SD =  15.04) [44] and Central Sensitization Score (CSS) of 54.12 (SD =  7.53) [46, 47]. Among medications taken by participants, 31% were prescribed medications specifically for enhancing sleep. Participants average Medication Quantification Scale Score III [48] was 10.74 (SD = 13.43). A complete detailing of participants sociodemographic and clinical characteristics is provided in Table 2.

**Table 2:** Overview of study cohort demographics.

Number of participants	Total N (%) 16 (100)
**Sociodemographic variables**	
**Age** (**years**)**, median** (**IQR**)	49.5 (33.0, 56.25)
**Sex, N** (**%**)	
Female	14 (87.5)
Male	2 (12.5)
**Race, N** (**%**)	
White/Caucasian	16 (100)
**Ethnicity, N** (**%**)	
Hispanic or Latino	0 (0)
Not Hispanic or Latino	16 (100)
Unknown	0 (0)
Not reported	0 (0)
**Body Mass Index, median** (**IQR**)	29.0 (24.0, 30.8)
**Annual Household Income** $9,999 or less	0 (0)
$10,000 to $19,999	1 (6.25)
$20,000 to $29,999	1 (6.25)
$30,000 to $39,999	0 (0)
$40,000 to $49,999	1 (6.25)
$50,000 to $59,999	0 (0)
$60,000 to $69,999	1 (6.25)
$70,000 to $79,999	2 (12.5)
$80,000 to $89,999	0 (0)
$90,000 or more	8 (50)
Choose not to answer	2 (12.5)
**Education**	
High School/GED	3 (18.75)
Some College	2 (12.5)
College	8 (50)
Masters	3 (18.75)
**Clinical variables**	**Mean** (**SD**)
Duration of pain (years)	9.99 (8.73)
Severity of pain (NRS)	6.38 (2.03)
**Quality of life (SF-36)**	
Physical functioning	28.44 (11.65)
Role limitations due to physical health	1.56 (6.25)
Role limitations due to emotional problems	41.67 (43.03
Energy/fatigue	18.44 (12.48)
Emotional well-being	57.50 (15.65)
Social functioning	39.06 (29.54)
Pain	30.62 (15.04)
General health	36.25 (14.20)
Central sensitization score (CSS)	54.12 (7.53)
**Medications**	**Mean** (**SD**)
**Medication Quantification Scale Score III**	
Total	10.74 (13.43)
Nonsteroidal Anti-inflammatory Drugs (NSAIDs)	3.61 (6.85)
Muscle relaxants	1.92 (3.58)
Neuropathic Pain	3.32 (5.73)
Benzodiazepines	1.46 (2.41)
Opioids	0.42 (1.70)
	**N** (**%**)
Patients prescribed sleep enhancing medications	5 (31.25)

### Tudor-Locke sensitivity analysis

The TLO set for our chronic pain population featured a *Bedtime Definition* of 5 minutes, a *Wake Time Definition* of 25 minutes, a *Min Sleep Period Length* of 160 minutes, and a *Minimum Non-Zero Epochs* of 5 minutes. Compared to TLD, TLO revised the default *Wake Time Definition* from 10 to 25 minutes and *Minimum Non-Zero Epochs* from 0 to 5 minutes. TLD achieved a Percentage of Agreement of 50% with the sleep logs, while TLO achieved 67%. An overview of the sensitivity analysis is provided in Section S2 of the Supplementary Materials along with the S1 Dataset containing results for every parameter set evaluated. On average, *Wake Time Definition* had the greatest impact on Percentage of Agreement, with lower values of *Wake Time Definition* decreasing Percentage of Agreement.

ActiGraph’s own sleep period detection algorithm achieved 56% agreement with sleep logs, outperforming the TLD algorithm (50% agreement), yet, was surpassed by the TLO algorithm (67% agreement). Given the superior agreement observed by TLO, we omitted ActiGraph’s custom algorithm from further analysis and focused solely on the TL algorithm.

### Comparison between default and optimized Tudor-Locke parameters

#### Sleep period detection.

The TL algorithm occasionally failed to detect a sleep period entirely, despite a sleep period being recorded in the participant’s sleep log for that night. For the TLD algorithm, 11 days were missed (10.2% of all days), spanning across 6 participants (37.5% of all participants). Among these 6 participants, an average of 1.83 (SD: 0.98) days were missed. For the TLO algorithm, 3 days were missed (2.8% of all days), across 3 participants (18.75% of all participants), with one night missed for each participant.

#### Sleep Measures.

The first analysis compared sleep measures between SL and TLD, and SL and TLO, using Extended Bland-Altman plots. Summary statistics from these plots are presented in Table 3, with plots for each sleep measure available in Section S3 of the Supplementary Materials. Overall, stronger agreement was seen between SL and TLO compared to SL and TLD. When comparing SL and TLD, proportional biases were observed for *Efficiency*, *Wake After Sleep Onset* (*WASO*), *Number of Awakenings* and *Movement Index*. In these instances, the difference between TL and SL increased as the average magnitude increased. However, these biases were not present between SL and TLO. For *Average Awakening Length*, a similar proportional bias was observed for both the SL and TLD comparison, and the SL and TLO comparison, respectively.

**Table 3:** Mean Bias and 95% Limits of Agreement derived from Bland-Altman are presented for each sleep measure. Comparisons are made between Tudor-Locke with default parameters and sleep logs, as well as Tudor-Locke with optimized parameters and sleep logs, with sleep logs serving as the “gold-standard” measure.

Sleep measure	Mean Bias (95% Limits of Agreement)	
	Tudor-Locke, Default	Tudor-Locke, Optimized
Latency	-10.5 (-36.5, 15.5)	-17.1 (-129.6, 95.4)
Efficiency (%)	12.6 (-4.0, 29.3)	4.3 (-17.0, 25.6)
Total Minutes in Bed	-139.9 (-574.2, 294.4)	36.2 (-341.3, 413.7)
Total Sleep Time (TST)	-49.9 (-395.0, 295.2)	50.7 (-170.9, 272.4)
Wake After Sleep Onset (WASO)	-79.3 (-220.4, 61.9)	-3.0 (-188.8, 182.8)
Number of Awakenings	-8.3 (-24.3, 7.6)	3.2 (-19.6, 25.9)
Average Awakening Length	-2.9 (-10.2, 4.3)	-1.1 (-7.8, 5.7)
Movement Index	-8.3 (-23.8, 7.1)	-1.2 (-23.4, 21.0)
Fragmentation Index	-3.8 (-18.1, 10.5)	-0.7 (-14.2, 12.9)
Sleep Fragmentation Index	-12.1 (-36.6, 12.4)	-1.9 (-31.6, 27.8)

**Fig 1:**
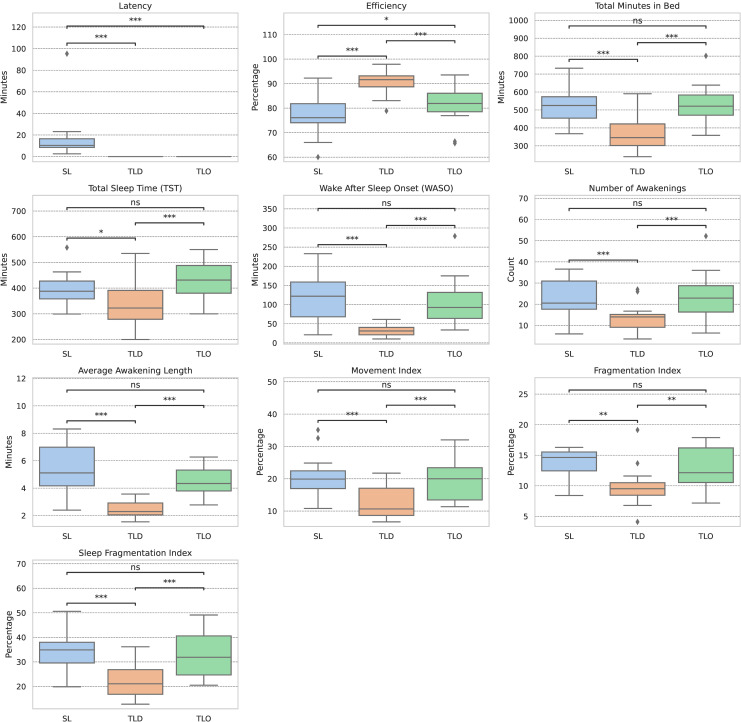
Boxplots detailing the distribution of participants mean sleep measures, stratified by each sleep period detection method: participant’s sleep logs (SL), the Tudor-Locke algorithm with default parameters (TLD), and the Tudor-Locke algorithm with optimized parameters (TLO). [49] Results of Wilcoxon Signed-Rank Test comparisons are included with significance values indicated as ns: 0.05 *< * P ≤  1.00; * : 0.01 *< * P ≤  0.05; **. 0.001 *<* P *≤* 0.01; ***: P *≤* 0.001. Note: this test was not performed for TLD vs TLO on the *Latency* measure as, per the Tudor-Locke algorithm, this measure always results in zero. Therefore, the test cannot be conducted.

In the second analysis, participant-level means were computed for each sleep measure. Wilcoxon Signed-rank tests were used to compare distributions between SL and TLD, SL and TLO, and TLD and TLO, respectively. Results are provided in Fig 1 as boxplots accompanied by the pair-wise comparisons (exact values for the Wilcoxon tests are available in Section S4 of the Supplementary Materials, while deidentified, aggregated sleep measures are available in the S2 Dataset). Statistically significant differences were observed between SL and TLD for all sleep measures, as well as between TLD and TLO. Statistically significant differences were observed between SL and TLO only for *Latency* (P *< * 0.001) and *Efficiency* (P =  0.025).

Finally, the means and standard deviations of each sleep measure across each detection method, are provided in Section S5 of the Supplementary Materials. For the supplementary analysis comparing sleep measures when stratified by participants age: ≤ 50 years and > 50 years, no significant differences in sleep measures were observed between these groups. These results were found to be reasonably consistent with observations from large scale studies [50]. A complete detailing of this analysis is provided in Section S6 of the Supplementary Materials.

## Discussion

The TL algorithm provides versatility in detecting sleep periods in accelerometry data through its adjustable parameters, however, the default settings may not suit all populations. Initially validated on a cohort of 30 fourth-grade children, its applicability to chronic pain sufferers with disrupted sleep patterns remains unclear [27, 28]. Moreover, the initial validation utilized waist-worn accelerometers [27], raising concerns about its validity for wrist-worn devices, which are generally preferred for sleep detection [51]. This limited understanding of the algorithm’s broader applicability highlights the need to reassess parameter values in different populations to ensure valid results.

Our sensitivity analysis found an optimal parameter set for our chronic pain population, where increasing the *Wake Time Definition* significantly improved agreement with sleep logs. This more conservative definition may better suit chronic pain patients who experience higher restlessness, preventing false identification of sleep period endings [7].

For *Minimum Non-Zero Epochs*, our optimized value of 5 epochs offered a negligible 0.01% improvement in agreement over the default of 0 epochs, assuming other parameters were optimized. Despite its limited impact in our setting—where participants consistently wore their devices at night—this parameter is crucial for analyzing 24-hour sleep-wake cycles, particularly in distinguishing sleep from non-wear periods, a known limitation of the Cole-Kripke algorithm [52].

When comparing sleep measures, TLO showed stronger agreement with SL than TLD, which is expected since TLO’s parameters were optimized to agree with SL. Instead, this comparison highlights the discrepancies between TLD and SL, indicating the default parameters are inadequate for this cohort of chronic pain patients.

### Comparison with prior work

To contextualize our resulting sleep measures, we have compared them with several prior studies examining similar cohorts and different sleep detection modalities, an overview is provided in Table 4.

**Table 4:** A comparison of sleep measures derived from Tudor-Locke with optimized parameters (TLO) to three previous studies: an accelerometry study featuring a chronic pain cohort (column ACC, Andrews et al. [22]), a polysomnography (PSG) meta-analysis of chronic pain cohorts (column PSG MA, Bjurstrom et al. [53]), and a nationally representative accelerometry study of older adults (column NSHAP, Kurina et al. [54]). We present the mean sleep measures for our cohort along with standard deviation (SD) and standard error (SE) to facilitate comparisons with the other studies.

Sleep Measure	Our Cohort			ACC	PSG MA	NSHAP
	Mean	(SD)	(SE)	Mean (SD)	Mean (SD)	Mean (SE)
Latency	0	(0)	(0)			
Efficiency (%)	81.2	(7.4)	(1.85)		75 (14)^a^ to 93 (10)^b^	82.4 (0.53)
Total Minutes in Bed	537.98	(102.23)	(25.56)	442.2 (115.8)		504 (4.2)
Total Sleep Time (TST) [minutes]	430.48	(71.22)	(17.8)		288 (71)^a^ to 475 (59)^c^	432.6 (3.3)
Wake After Sleep Onset (WASO)	107.49	(58.52)	(14.63)		19 (10)^d^ to 64 (44)^a^	38.8 (1.2)
Number of Awakenings	24.48	(10.84)	(2.71)	6.63 (4.39)		
Average Awakening Length	4.45	(1.05)	(0.26)	6.48 (4.99)		
Movement Index (%)	19.85	(7.1)	(1.77)			
Fragmentation Index (%)	12.94	(3.57)	(0.89)			14.4 (0.33)
Sleep Fragmentation Index (%)	32.79	(9.88)	(2.47)			

^a^Mork et al. [55]; ^b^Horne et al. [56]; ^c^Shaver et al. [57]; ^d^Gonzalez et al. [58].

First, we compared our sleep measures with a similar study utilizing accelerometry to measure sleep in chronic pain patients. Andrews, et. al, examined 50 older adults (mean age =  54.22, SD =  10.68) with chronic pain [22]. Sleep measures were derived using GT3X ActiGraph accelerometers and participant’s sleep logs. Referring to Table 4, *Total Minutes in Bed* and *Average Awakening Length* were found to be reasonably consistent between the two studies. However, participants in our cohort awoke, on average, 17.85 more times per night. This discrepancy may be due in part to our cohort being predominantly women (87.5%), as Andrews, et. al, reported that their female participants had more awakenings, on average, compared to male participants.

Second, we compared our accelerometry-derived sleep measures to PSG, focusing on a meta-analysis of PSG studies (n =  19) conducted in fibromyalgia and chronic widespread pain populations [53]. Referring to Table 4, *TST* and *Efficiency* fell within ranges reported by PSG, however, *WASO* was higher in our cohort.

Finally, we compared our results to the National Social Life, Health, and Aging Project (NSHAP), a nationally representative cohort of 739 older adults (ages 62-90) monitored using ActiWatch Spectrum accelerometers [54]. This comparison assessed the similarity between our cohort and the general older adult population. As shown in Table 4, our cohort’s sleep measures were reasonably consistent, except for higher *WASO*.

Overall, these comparisons suggest many of the sleep measures observed in our study are within the range of those reported in similar populations and when gathered using PSG. Metrics representing amount of time spent awake through the sleep session (*WASO* and *Number of Awakenings*), however, were higher in our study which may be due to restlessness/limb movement during sleep being misclassified as awake time with actigraphy.

### Limitations

In our study, TL parameter optimization was based on self-reported sleep logs, which are susceptible to recall bias [59], and was constrained by a small sample size of 16 participants. Therefore, further sensitivity analyses with larger samples and different platforms are needed to ensure reliable results. Although a revised TL algorithm was released in 2018 [60], we used the original version, as it was the version implemented in ActiLife at the time of writing [31].

## Conclusion

Automated sleep detection via accelerometry is inherently difficult. While the TL algorithm provides investigators with a variety of useful sleep measures based on the sleep periods it identifies, the accuracy of the algorithm depends on its parameter settings. Having been validated on healthy children and young adults, the algorithm’s default parameters may not be suitable for populations who experience abnormal or disruptive sleep patterns, such as those with chronic pain. As such, sensitivity analyses are needed to reevaluate these parameters for broader applicability. This manuscript presents a sensitivity analysis that refines the TL parameter set for a cohort of adults with chronic pain. The optimized parameter set provides researchers studying chronic pain populations with a more reliable starting point for identifying sleep periods, thereby enhancing the accuracy of the sleep metrics it derives. This optimization enables more accurate assessments of outcomes and intervention effectiveness pertaining to sleep amongst this population, broadening the algorithms applicability while improving clinical understanding and care.

## Supporting information

S1 File Supplementary materials. PDF containing supplementary analyses, tables, and figures.
(PDF)

S1 Dataset Sensitivity analysis. Complete results of the sensitivity analysis.
(CSV)

S2 Dataset Participant’s deidentified, aggregated sleep measures as generated by the three different sleep detection methods. (XLSX)

## Abbreviations:

IPMP: Interdisciplinary Pain Management Program;
PSG: Polysomnography;
SL: Sleep logs;
TL: Tudor-Locke;
TLD: Tudor-Locke with default parameters;
TLO: Tudor-Locke with optimized parameters;
TST: Total Sleep Time;
WASO: Wake After Sleep Onset.
