# Supplementary material for: Optimizing an automated sleep detection algorithm using wrist-worn accelerometer data for individuals with chronic pain
Source: PLoS One. 2025 Apr 4;20(4):e0319348. doi: 10.1371/journal.pone.0319348 (PMC11970680; doi:10.1371/journal.pone.0319348)
Supplement: S1 File — PDF containing supplementary analyses, tables, and figures. (PDF) [file pone.0319348.s001.pdf]

S1 Consistency Across Missing Data Strategies

This analysis compared sleep measures across two missing data strategies: complete-case analysis and zero-imputation. Comparisons were made using the performance metrics: Mean Squared Error (MSE), Root Mean Squared Error (RMSE) and Mean Absolute Error (MAE), measuring the discrepancy between TLD vs. SL and TLO vs. SL, respectively. SL served as "ground truth". These metrics were computed within each participant and then averaged across participants.

Results are provided in Table S1, with the better score appearing in bold. Overall, higher agreement levels were observed between TLO and SL compared to TLD and SL for all measures, regardless of missing data strategy. The sleep measure *Latency*, was an exception to this. However, recall the TL algorithm will always produce a *Latency* of zero, regardless of parameter settings.

Table S1: Measures of MSE, RMSE, and MAE showing the distance between Tudor-Locke with default parameters (TLD) and sleep logs (SL), as well as distance between Tudor-Locke with optimized parameters (TLO) and sleep logs. Results are shown for two missing data strategies: zero-imputation and complete-case analysis.

		Zero-Imputation			Complete-case Analysis		
**Sleep measure**	**TL Method**	**MSE**	**RMSE**	**MAE**	**MSE**	**RMSE**	**MAE**
Latency	Default	3326.86	29.16	16.56	**266.73**	**14.49**	**10.31**
	Optimized	3326.86	29.16	16.56	3331.35	29.34	16.83
Efficiency	Default	723.86	23.42	18.51	231.91	14.3	12.74
	Optimized	**395.49**	**16.14**	**10.68**	**133.1**	**10.5**	**8.19**
Total Minutes in Bed	Default	86546.01	270.61	230.82	68837.74	244.37	207.71
	Optimized	**41256.39**	**176.67**	**139.26**	**37494.29**	**165.74**	**131.99**
Total Sleep Time (TST)	Default	42493.58	183.24	147.22	33034.06	162.02	129.48
	Optimized	**20520.19**	**118.41**	**87.95**	**15346.24**	**103.11**	**78.74**
Wake After Sleep Onset (WASO)	Default	12607.17	99.1	83.84	11612.87	95.46	80.86
	Optimized	**8266.99**	**78.47**	**61.89**	**8727.31**	**79.88**	**64.02**
Number of Awakenings	Default	175.63	11.83	10.05	141.77	10.89	9.32
	Optimized	**130.34**	**8.51**	**6.63**	**143.34**	**8.73**	**6.93**
Average Awakening Length	Default	24.47	4.36	3.42	23.06	3.99	3.12
	Optimized	**13.31**	**3.05**	**2.14**	**13.37**	**3.05**	**2.16**
Movement Index	Default	218.94	13.14	10.57	130.34	10.29	8.67
	Optimized	**123.51**	**9.75**	**7.01**	**125.2**	**9.85**	**7.15**
Fragmentation Index	Default	81.53	8.41	6.25	66.35	7.44	5.68
	Optimized	**45.49**	**5.62**	**3.92**	**46.84**	**5.71**	**4.04**
Sleep Fragmentation Index	Default	472.28	19.72	15.86	301.26	16.02	13.25
	Optimized	**221.23**	**12.88**	**9.6**	**224.66**	**13.03**	**9.81**

S2 Sensitivity Analysis

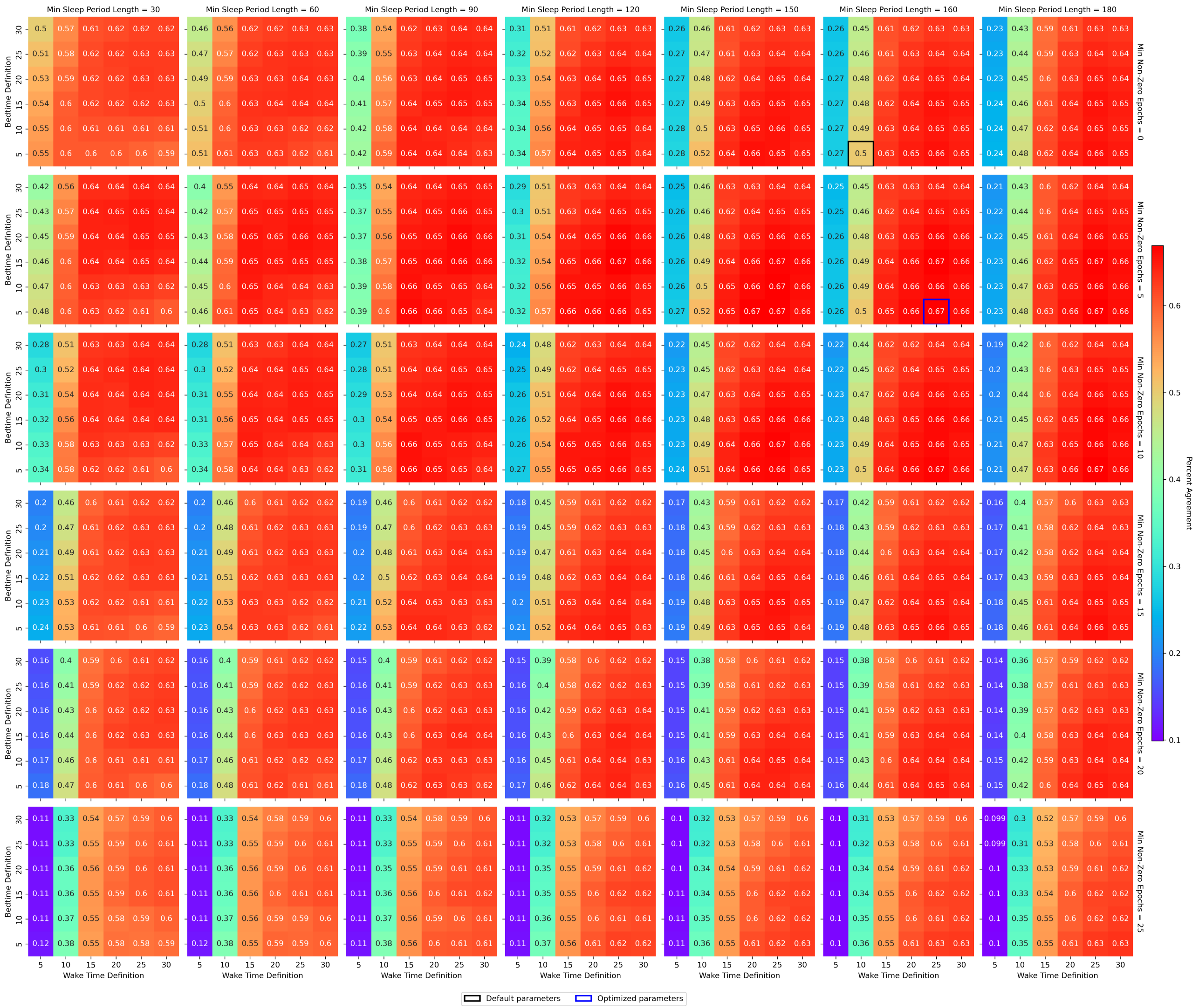

Figure S1: A grid of heatmaps detailing the complete results of the sensitivity analysis. Values indicate the Percentage of Agreement between sleep periods identified by the Tudor-Locke algorithm and sleep periods obtained from participants sleep logs, for each parameter set tested. Each column of the grid represents a different *Min Sleep Period Length* tested, while rows indicate the *Min Non-Zero Epochs* value tested. Within each heatmap, the X-axis indicates the *Wake Time Definition* tested, while the Y-axis indicates the *Bedtime Definition* tested. Each parameter value is represented in minutes. The box outlined in black indicates the agreement when the Tudor-Locke algorithm's default parameter sets were utilized, while the blue box indicates that maximum agreement achieved.

S3 Extended Bland-Altman Plots

Extended Bland-Altman plots detailing agreement for 1) Sleep measures derived from the Tudor-Locke algorithm with default parameters compared to sleep logs (SL vs TLD) and 2) Sleep measures derived from the Tudor-Locke algorithm with optimized parameters compared to sleep logs (SL vs TLO). Dots with the same color reflect data points belonging to the same individual.

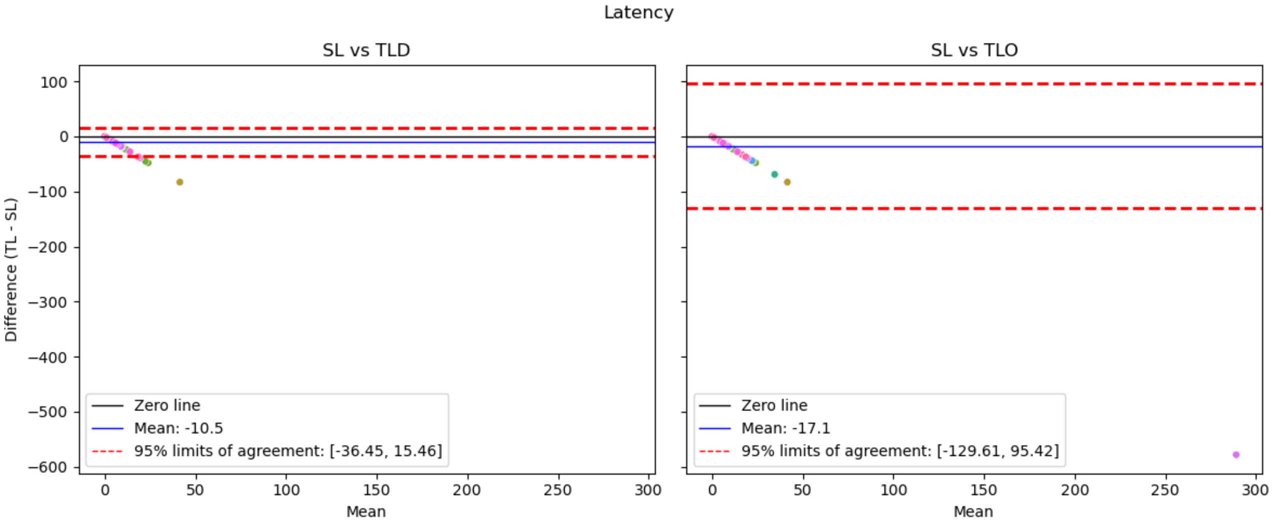

Figure S2: The TLD approach underestimated latency by 10.5 minutes, while the TLO approach underestimated latency by 17.1 minutes. Note: the significant proportional bias observed is a result of the TL algorithm always producing a latency of 0, regardless of its parameter values.

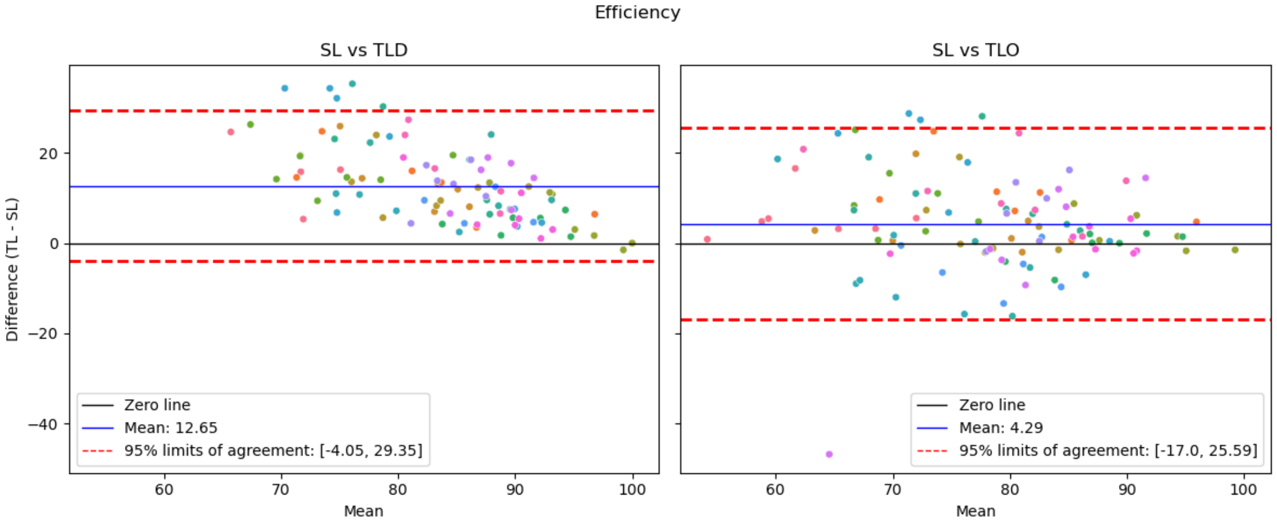

Figure S3: The TLD approach overestimated efficiency by 12.65%, while the TLO approach overestimated efficiency by 4.3%. A proportional bias was observed for TLD, but appeared absent for TLO.

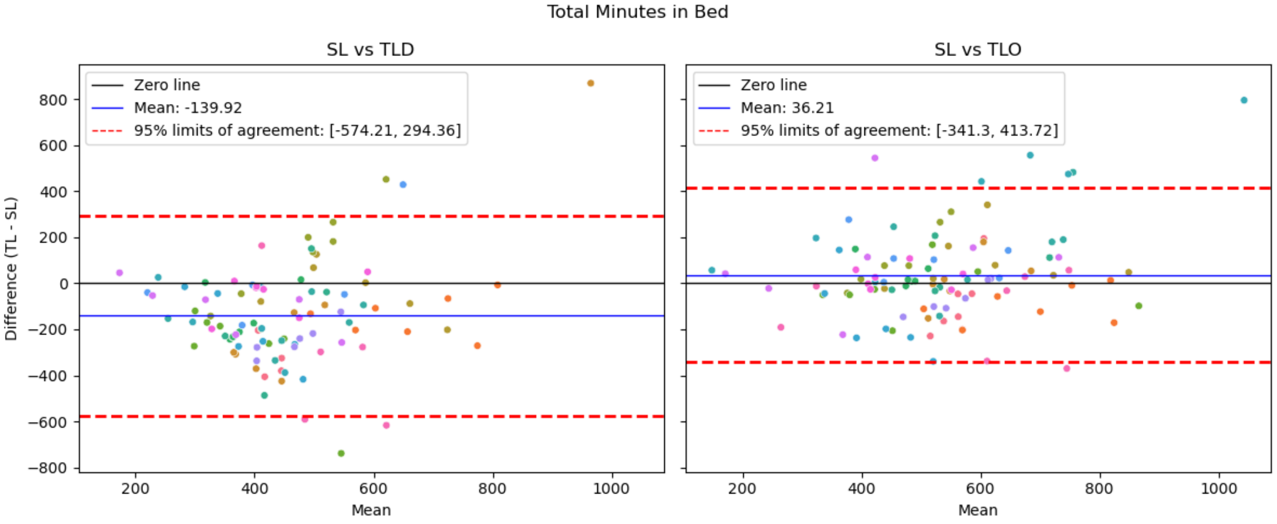

Figure S4: The TLD approach underestimated total minutes in bed by 139 minutes, while the TLO approach overestimated total minutes in bed by 36 minutes.

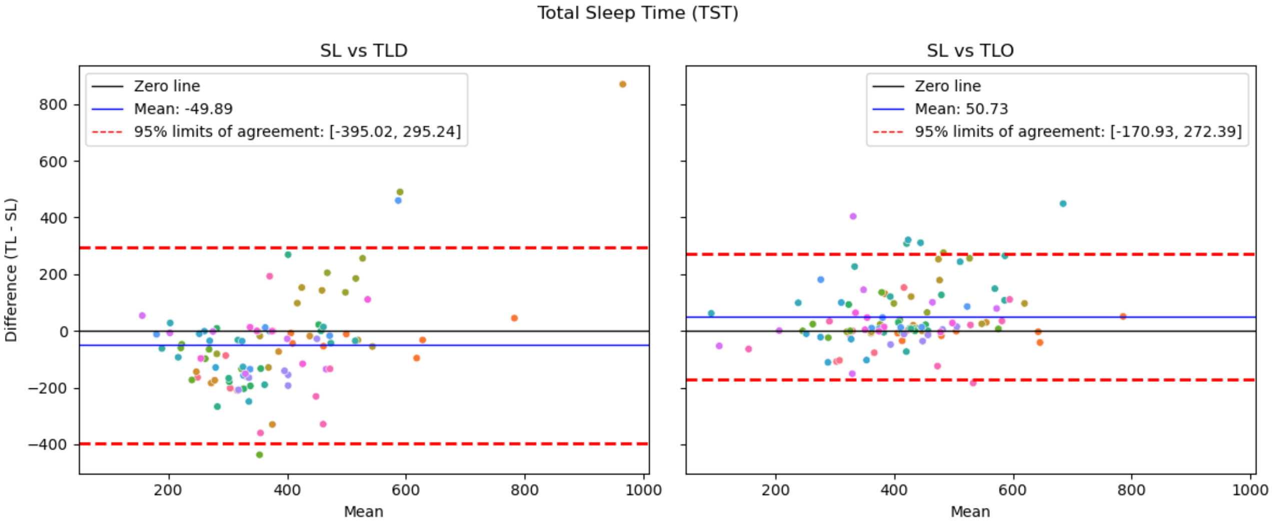

Figure S5: The TLD approach underestimated total sleep time by 49 minutes, while the TLO approach overestimated total sleep time by 50 minutes.

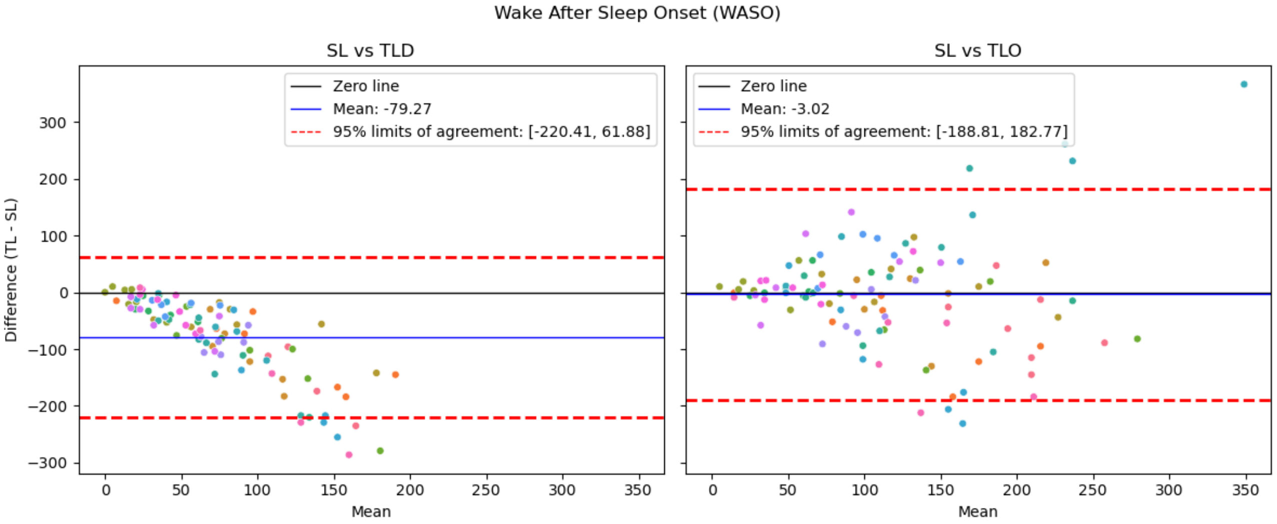

Figure S6: The TLD approach underestimated wake after sleep onset by 79 minutes, while the TLO approach underestimated wake after sleep onset by 3 minutes. A proportional bias was observed for TLD, but appeared absent for TLO.

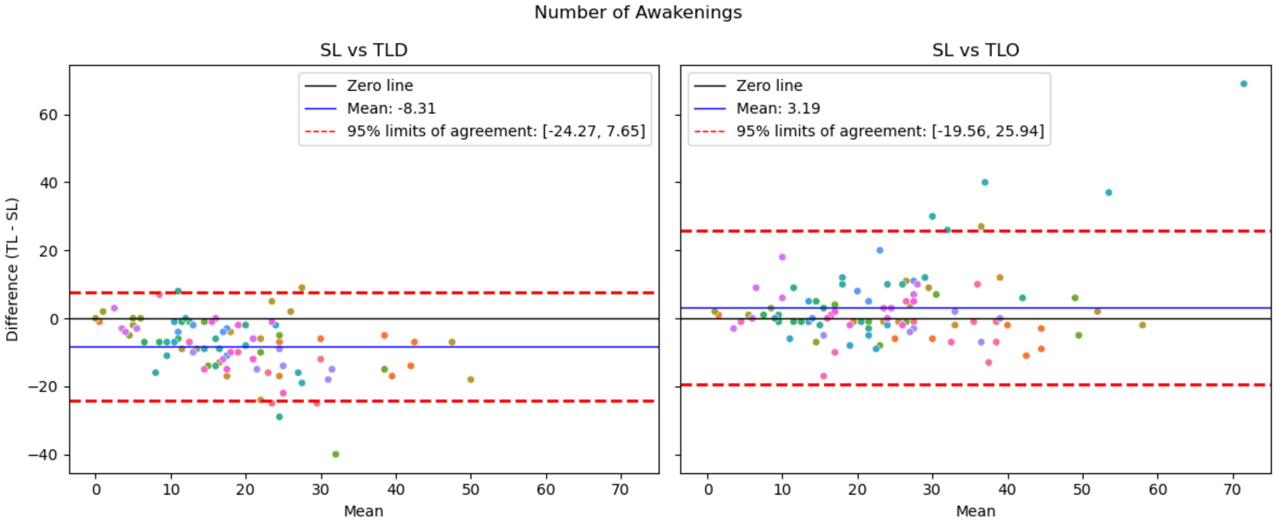

Figure S7: The TLD approach underestimated the number of awakenings by 8, while the TLO approach overestimated the number of awakenings by 3. A proportional bias was observed for TLD, but appeared absent for TLO.

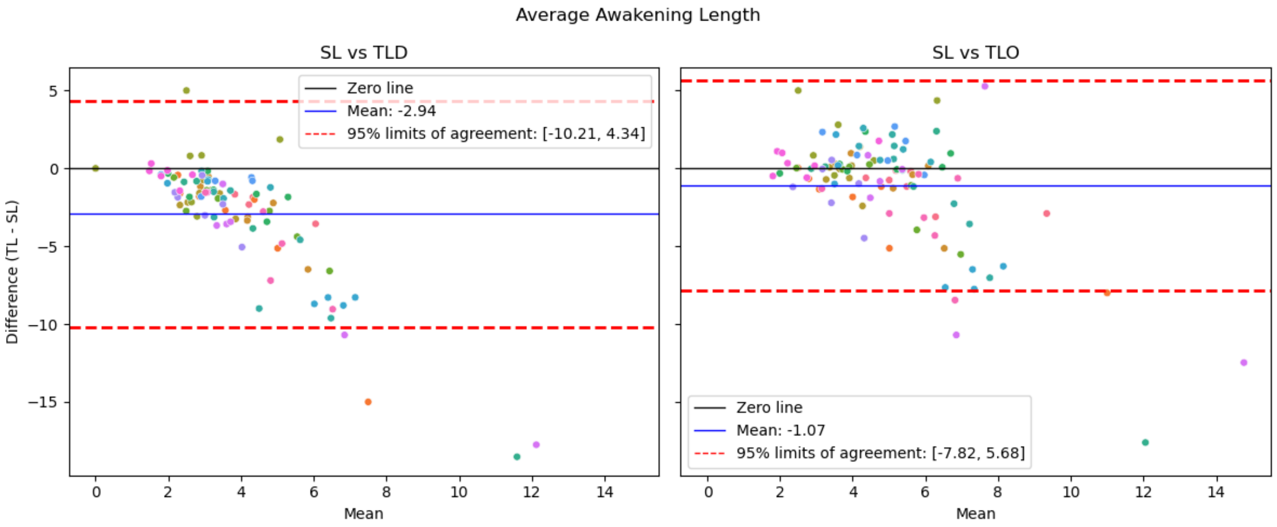

Figure S8: The TLD approach underestimated average awakening length by 3 minutes, while the TLO approach underestimated average awakening length by 1 minute. A proportional bias was observed for TLD and TLO.

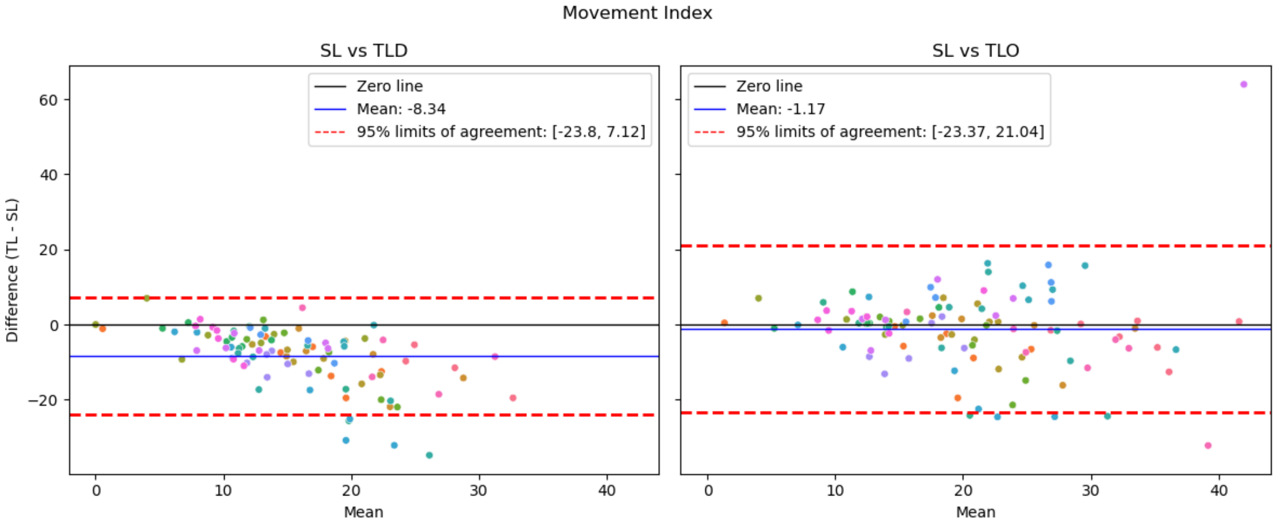

Figure S9: The TLD approach underestimated the Movement Index by 8.34%, while the TLO approach underestimated the Movement Index 1.17%. A proportional bias was observed for TLD, but appeared absent for TLO.

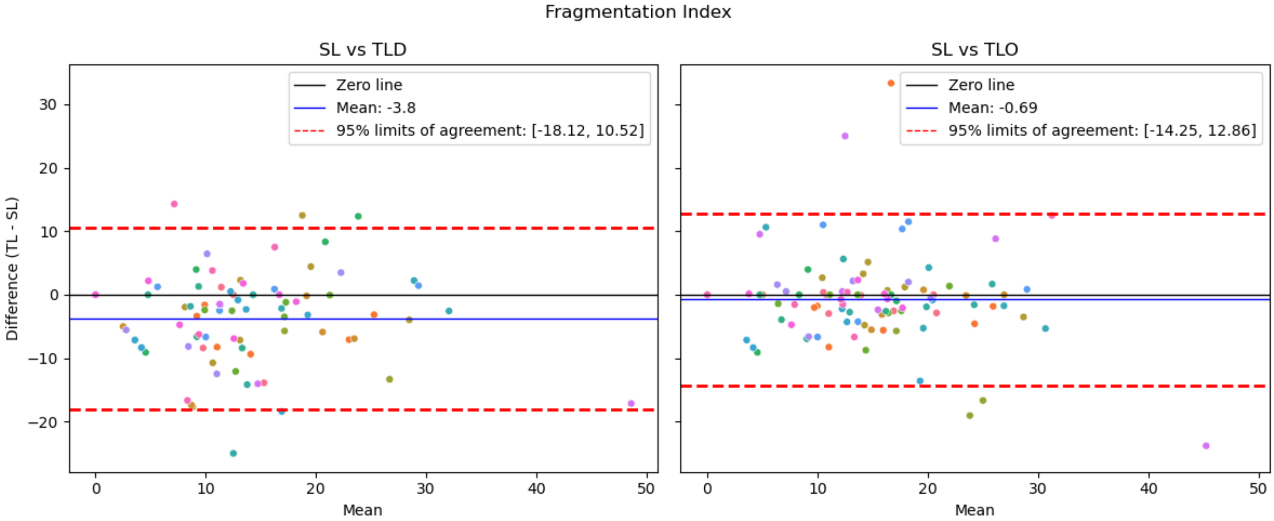

Figure S10: The TLD approach underestimated the Fragmentation Index by 3.8%, while the TLO approach underestimated Fragmentation Index by 0.69%.

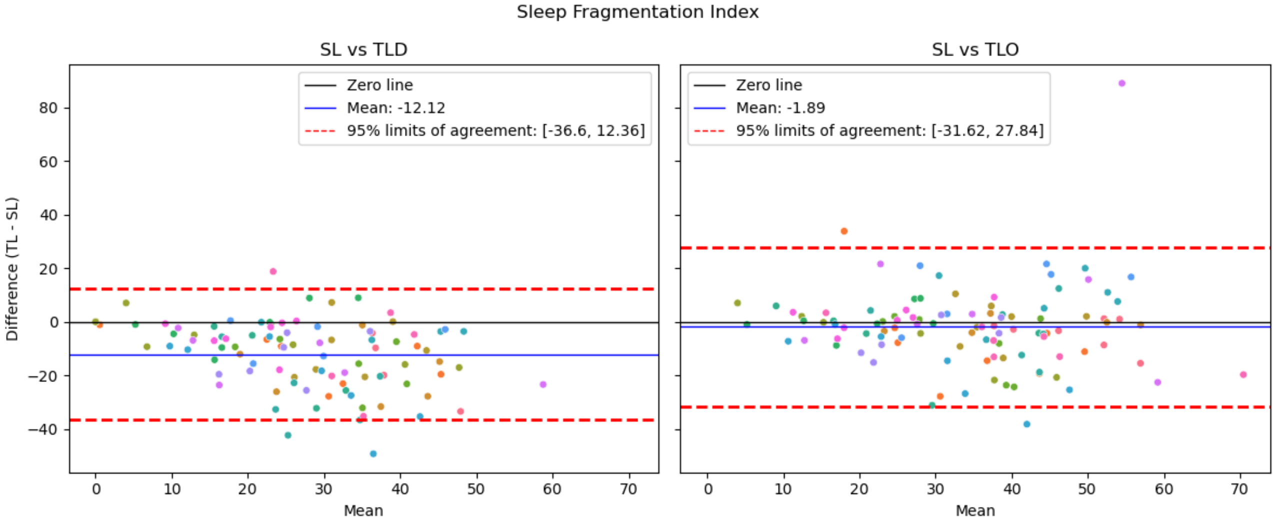

Figure S11: The TLD approach underestimated the Sleep Fragmentation Index by 12.12%, while the TLO approach underestimated the Sleep Fragmentation Index by 1.89%.

S4 Wilcoxon Signed-Rank Tests

Table S2: Results of the Wilcoxon Signed-Rank Tests measuring whether significant differences in sleep measures existed between the three sleep detection algorithms: sleep logs (SL), Tudor-Locke algorithm with default parameters (TLD), and the Tudor-Locke algorithm with optimized parameters (TLO). P-values < 0.05 suggest the two approaches yielded statistically significant differences.

	**Wilcoxon Statistic (P-Value)**		
**Sleep measure**	**SL vs. TLD**	**SL vs. TLO**	**TLD vs. TLO**
Latency	0 (<0.001)	0 (<0.001)	NA
Efficiency	0 (<0.001)	25 (0.025)	0 (<0.001)
Total Minutes in Bed	8 (0.001)	66 (0.940)	1 (<0.001)
Total Sleep Time (TST)	20 (0.011)	33 (0.074)	6 (<0.001)
Wake After Sleep Onset (WASO)	0 (<0.001)	56 (0.562)	0 (<0.001)
Number of Awakenings	0 (<0.001)	54 (0.495)	0 (<0.001)
Average Awakening Length	3 (<0.001)	34 (0.083)	0 (<0.001)
Movement Index	0 (<0.001)	60 (0.706)	0 (<0.001)
Fragmentation Index	11 (0.002)	46 (0.274)	13 (0.003)
Sleep Fragmentation Index	2 (<0.001)	53 (0.464)	0 (<0.001)

S5 Descriptive Analysis

Table S3: Mean (SD) values for sleep measures, stratified by each sleep period detection method: participant's sleep logs (SL), the Tudor-Locke algorithm with default parameters (TLD), and the Tudor-Locke algorithm with optimized parameters (TLO).

	**Mean (SD)**		
**Sleep measure**	**Sleep Logs**	**Tudor-Locke, Default**	**Tudor-Locke, Optimized**
Latency	16.56 (21.78)	0.0 (0.0)	0.0 (0.0)
Efficiency	76.79 (8.1)	90.26 (4.91)	81.2 (7.4)
Total Minutes in Bed	519.31 (96.98)	373.05 (103.83)	537.98 (102.23)
Total Sleep Time (TST)	394.12 (65.9)	339.88 (102.49)	430.48 (71.22)
Wake After Sleep Onset (WASO)	113.71 (55.18)	33.17 (15.48)	107.49 (58.52)
Number of Awakenings	22.0 (8.96)	13.45 (6.32)	24.48 (10.84)
Average Awakening Length	5.39 (1.85)	2.48 (0.6)	4.45 (1.05)
Movement Index	20.33 (6.81)	12.25 (4.58)	19.85 (7.1)
Fragmentation Index	13.69 (2.51)	9.87 (3.24)	12.94 (3.57)
Sleep Fragmentation Index	34.03 (8.26)	22.11 (6.69)	32.79 (9.88)

S6 Comparative Analysis

A comparison of sleep measures between participants less than 50 years of age ($n = 8$) and those 50 years of age or older ($n = 8$) was performed. The mean age of participants in the ≤50 group was 33.75 ($SD = 10.34$), while the mean age of participants >50 was 58.50 ($SD = 6.61$). Each group consisted of seven women and one man. Sleep measures were derived using the TLO approach and group-level comparisons were made using Wilcoxon signed-rank tests with a significance threshold of $P = 0.05$. No statistically significant differences were observed across the various sleep measures.

These observed differences are reasonably consistent with large scale averages among these age groups. A large scale study involving 1.1 million people from the Netherlands, United Kingdom and United States reported sleep measures stratified by age, specifically, *Time in bed*, *Total sleep time*, and *Sleep efficiency*. [1] On average, women ages 26-40 had a *Time in bed* of 486 minutes (SD = 48), *Total sleep time* of 438 minutes (SD = 60), and *Sleep efficiency* of 89% (SD = 10%). Women ages 41-65, on average, experienced a *Time in bed* of 474 minutes (SD = 54), *Total sleep time* of 426 minutes (SD = 66), and *Sleep efficiency* of 89% (SD = 10%).

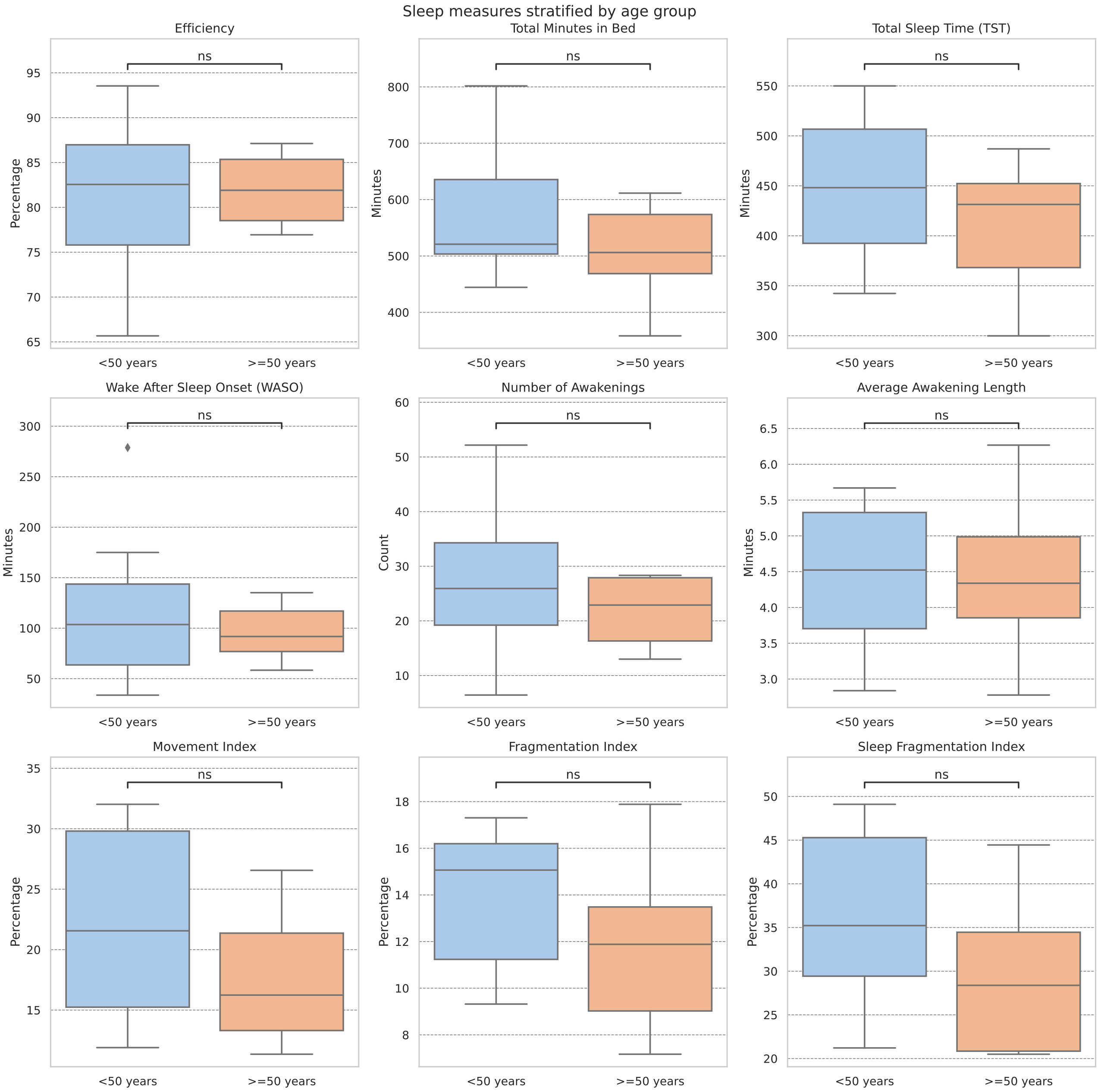

Figure S12: Boxplots detailing the distribution of participants mean sleep measures, stratified by two age groups: ≤50 years and >50 years). [2] Results of Wilcoxon Signed-Rank Test comparisons are included with significance values indicated as ns: 0.05 < P ≤ 1.00; *: 0.01 < P ≤ 0.05; **: 0.001 < P ≤ 0.01; ***: P ≤ 0.001.

References

[1] D. Kocevska, T. S. Lysen, A. Dotinga, M. E. Koopman-Verhoeff, M. P. Luijk, N. Antypa, N. R. Biermasz, A. Blokstra, J. Brug, W. J. Burk, *et al.*, "Sleep characteristics across the lifespan in 1.1 million people from the netherlands, united kingdom and united states: a systematic review and meta-analysis," *Nature human behaviour*, vol. 5, no. 1, pp. 113–122, 2021.

[2] F. Charlier, M. Weber, D. Izak, E. Harkin, M. Magnus, J. Lalli, L. Fresnais, M. Chan, N. Markov, O. Amsalem, S. Proost, A. Krasoulis, getzze, and S. Repplinger, "Statannotations," Oct. 2022.
